# Safety and Immunogenicity of a Newcastle Disease Virus Vector-Based SARS-CoV-2 Vaccine Candidate, AVX/COVID-12-HEXAPRO (Patria), in Pigs

**DOI:** 10.1128/mBio.01908-21

**Published:** 2021-09-21

**Authors:** Jesús Horacio Lara-Puente, Juan Manuel Carreño, Weina Sun, Alejandro Suárez-Martínez, Luis Ramírez-Martínez, Francisco Quezada-Monroy, Georgina Paz-De la Rosa, Rosalía Vigueras-Moreno, Gagandeep Singh, Oscar Rojas-Martínez, Héctor Elías Chagoya-Cortés, David Sarfati-Mizrahi, Ernesto Soto-Priante, Constantino López-Macías, Florian Krammer, Felipa Castro-Peralta, Peter Palese, Adolfo García-Sastre, Bernardo Lozano-Dubernard

**Affiliations:** a Laboratorio Avi-Mex, S. A. de C. V. (Avimex), Iztapalapa, CDMX, Mexico; b Department of Microbiology, Icahn School of Medicine at Mount Sinai, New York, New York, USA; c The Tisch Cancer Institute, Icahn School of Medicine at Mount Sinai, New York, New York, USA; d Department of Medicine, Division of Infectious Diseases, Icahn School of Medicine at Mount Sinai, New York, New York, USA; e Global Health and Emerging Pathogens Institute, Icahn School of Medicine at Mount Sinai, New York, New York, USA; f Department of Pathology, Icahn School of Medicine at Mount Sinai, New York, New York, USA; g Unidad de Investigación Médica en Inmunoquímica, UMAE Hospital de Especialidades del Centro Médico Nacional Siglo XXI. Instituto Mexicano del Seguro Social (IMSS), Cuauhtémoc, CDMX, Mexico; h Diagnósticos Clínicos Veterinarios, S. A. de C. V. (DCV), Iztapalapa, CDMX, Mexico; i Consultora Mextrategy, S. A. S. de C. V., Benito Juárez, CDMX, Mexico; Ohio State University

**Keywords:** COVID-19, SARS-CoV-2, coronavirus vaccine, pig model, Newcastle disease virus, NDV, HexaPro, spike, prolines, pigs, vaccine

## Abstract

Vaccines against severe acute respiratory syndrome coronavirus 2 (SARS-CoV-2) were developed in record time and show excellent efficacy and effectiveness against coronavirus disease 2019 (COVID-19). However, currently approved vaccines cannot meet the global demand. In addition, none of the currently used vaccines is administered intranasally to potentially induce mucosal immunity. Here, we tested the safety and immunogenicity of a second-generation SARS-CoV-2 vaccine that includes a stabilized spike antigen and can be administered intranasally. The vaccine is based on a live Newcastle disease virus vector expressing a SARS-CoV-2 spike protein stabilized in a prefusion conformation with six beneficial proline substitutions (AVX/COVID-12-HEXAPRO; Patria). Immunogenicity testing in the pig model showed that both intranasal and intramuscular application of the vaccine as well as a combination of the two induced strong serum neutralizing antibody responses. Furthermore, substantial reactivity to B.1.1.7, B.1.351, and P.1 spike variants was detected. Finally, no adverse reactions were found in the experimental animals at any dose level or delivery route. These results indicate that the experimental vaccine AVX/COVID-12-HEXAPRO (Patria) is safe and highly immunogenic in the pig model.

## INTRODUCTION

The pandemic of the coronavirus disease 2019 caused by the severe acute respiratory syndrome coronavirus 2 (SARS-CoV-2) (1, 2) triggered unprecedented efforts in search of vaccines. Vaccines were developed at record speed, and several have already been authorized for use and are widely available in high-income countries (3). However, vaccine availability in low- and middle-income countries (LMICs) is insufficient, and producers of current vaccines cannot satisfy the global demand, as recently highlighted by the Pan-American Health Organization (4). It is therefore necessary to extend the portfolio of available safe and effective vaccines to enhance vaccination programs globally. In addition, while currently available mRNA, adenovirus-vectored, and inactivated vaccines are highly protective, they do not express fully optimized spike antigens and were also not designed to induce mucosal immunity (3).

We developed a vaccine candidate (AVX/COVID-12-HEXAPRO, Patria; currently in phase I clinical trials [NCT04871737]) based on a live Newcastle disease virus (NDV) vector which expresses a highly optimized version of the spike protein on its surface and in infected cells. The current vaccine candidate has been optimized based on earlier iterations of this concept (5, 6). NDV is an avian virus which does not replicate in mammalian cells efficiently but triggers an innate immune response which acts as a “self-adjuvant” (7–10). While different NDV strains can be highly pathogenic in chickens, live virus vaccines, including the LaSota strain, have been developed as avian vaccines against virulent NDV (8, 11). The vaccine candidate here is based on the LaSota vaccine strain, which is safe in poultry; NDV has also been shown to be a safe oncolytic agent in humans (12, 13). The vaccine is expressing a highly optimized version of the spike protein. The already very stable HexaPro construct (14), which is held in the prefusion conformation by the introduction of six prolines in regions that turn into helices during the fusion process, was further optimized by C-terminal fusion with the transmembrane domain and cytoplasmic tail of the NDV fusion protein to enhance incorporation into the viral particles.

Respiratory virus infections are typically acquired by exposure of the upper and/or lower respiratory tract. Mucosal immunity is the first line of defense in the upper respiratory tract and often neutralizes incoming virus before an infection can establish itself. Optimal induction of mucosal immunity requires mucosal delivery of antigens to induce tissue-resident B- and T-cells. None of the currently used SARS-CoV-2 vaccines are delivered via this route, and they therefore induce strong systemic but low mucosal immune responses.

We tested this vaccine candidate in the pig model, a large-animal model for safety and immunogenicity studies, via the intramuscular (IM) or intranasal (IN) route or a combination of the two. Here, we report results from these studies which formed the basis for the phase I clinical trial design (NCT04871737).

## RESULTS

### Vaccine candidate and experimental design.

**FIG 1:**
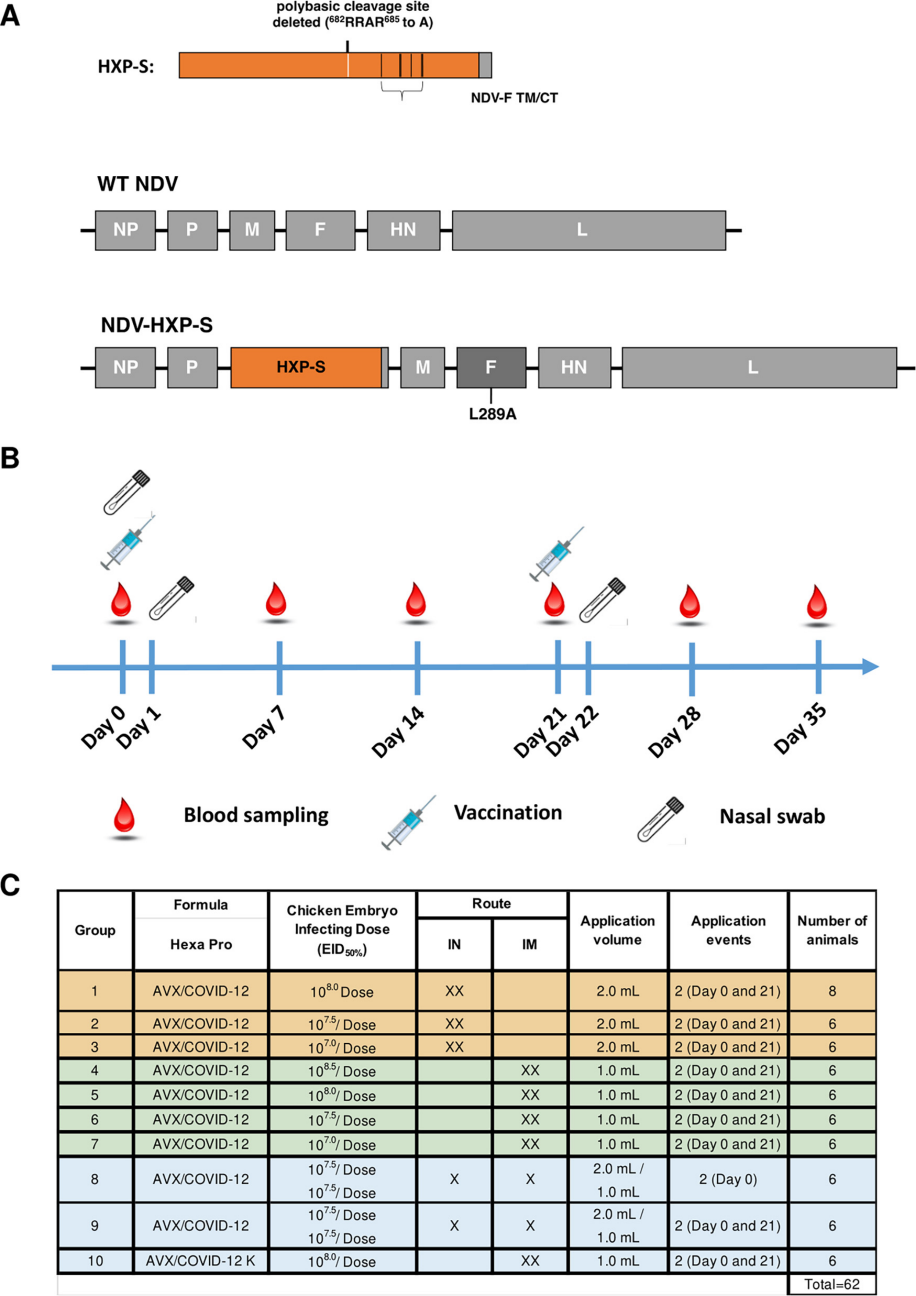
Schematic representation of AVX/COVID-12-HEXAPRO production and testing in pigs. Vector design is shown in panel A. The ectodomain of the spike protein was fused to the transmembrane domain and cytoplasmic tail (TM/CT) of the fusion (F) protein of the NDV by a short GGGGS linker to generate the S/F chimera. The polybasic cleavage site (RRAR) was eliminated by removing the three arginines (R). HEXAPRO stabilizing mutations were introduced into the ectodomain of spike. The construct is designated HXP-S. The nucleotide sequence of HXP-S was codon optimized for mammalian cell expression. The sequence was inserted between the P and M genes in the NDV genome (LaSota strain), which harbors an L289A mutation in the F protein. The experimental design is shown in panel B. Pigs were vaccinated with AVX/COVID-12-HEXAPRO on day 0 and day 21. Blood samples were collected at days 0, 7, 14, 21, 28, and 35 after the first vaccine dose administration. Nasal swabs were collected at days 0, 2, and 22 after the first vaccine dose administration. Distribution of groups is shown in panel C. Ten different groups were included in the study. Classification into IN-IN vaccinated animals (groups 1 to 3), IM-IM vaccinated animals (groups 4 to 7), and other vaccination regimens (groups 8 to 10) is shown. The corresponding dose, volume, and time points of vaccine administration are described. The sample number of pigs per group is indicated.

The vaccine was generated by engineering a HexaPro version (14) of the SARS-CoV-2 spike protein into the NDV LaSota strain. The HexaPro spike construct was further modified by C-terminal fusion with the transmembrane and C-terminal domains of the NDV fusion protein to enhance incorporation of the spike into the NDV particles (Fig. 1A). Vaccine viruses were manufactured in embryonated eggs at a pilot facility at Avimex (Mexico City, Mexico). Three- to 10-week-old specific-pathogen-free (SPF) piglets were vaccinated in a prime-boost regimen with a 21-day interval (Fig. 1B). Three groups of animals received the vaccine IN twice (target doses 10^8^, 10^7.5^, and 10^7^ 50% egg infectious doses [EID_50_]), four groups received the vaccine IM twice (target doses 10^8.5^, 10^8^, 10^7.5^, and 10^7^ EID_50_), one group received the vaccine IN and IM simultaneously without further boost (target dose 10^7.5^ EID_50_), one group received an IN prime followed by an IM boost (target dose 10^7.5^ EID_50_), and one group received two IM shots with inactivated vaccine plus water oil water (WOW) adjuvant equivalent to a 10^8^-EID_50_ target dose.

### Clinical observations and safety testing.

**FIG 2:**
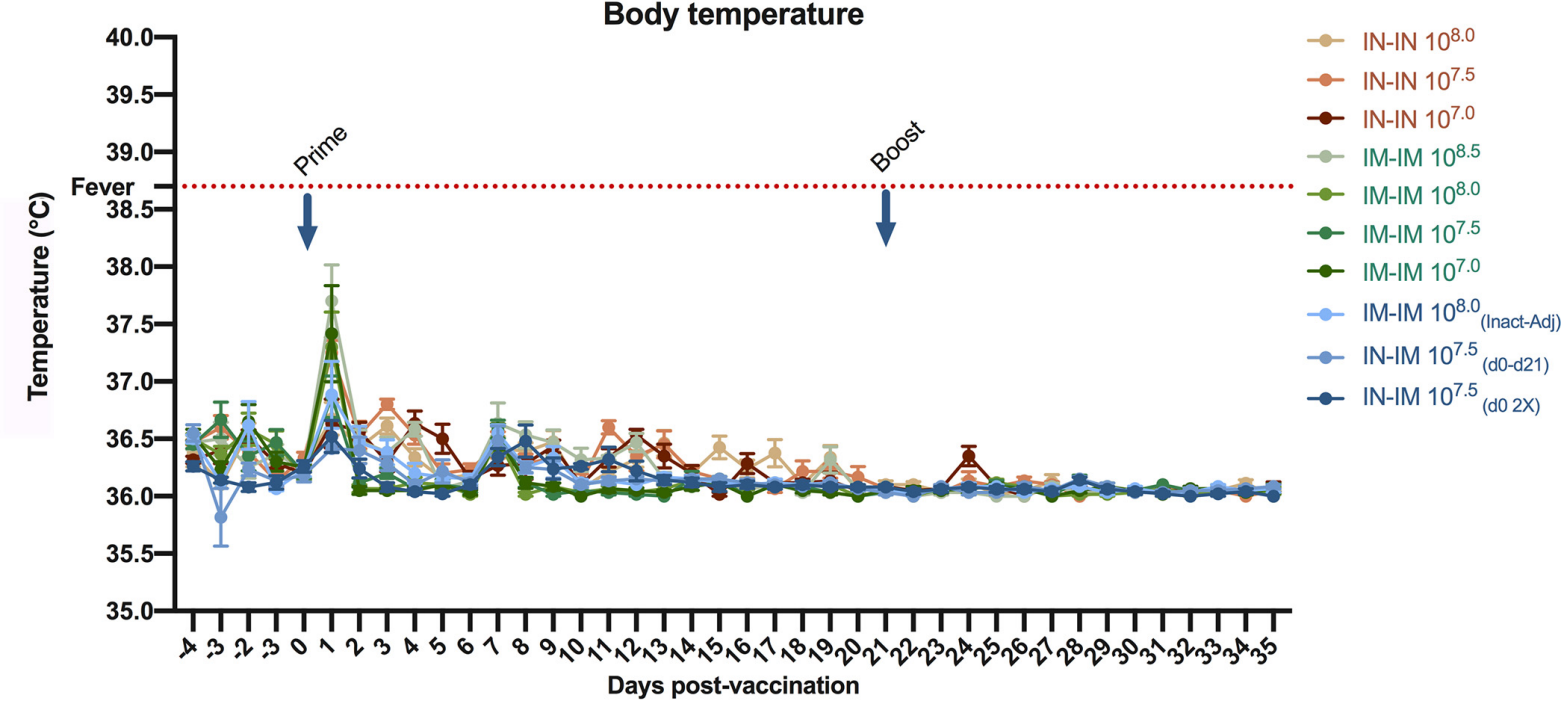
Kinetics of body temperature in AVX/COVID-12-HEXAPRO-vaccinated pigs. Temperature (°C) of pigs was measured daily 4 days before through 35 days after the vaccine administration. The reference value for fever in pigs (38.8°C) is indicated by the red dotted line. Vaccination time points (prime and boost) are indicated with blue arrows. Mean of daily temperature per group plus standard error of the mean (SEM) is shown.

The animals were acclimatized for 4 days before the start of the study. The animals were monitored once a day for clinical signs, from the beginning to the end of the study. Each active formulation was evaluated separately and simultaneously. As a substitute for reactogenicity, body temperature was reported daily throughout the course of the experiment. While transient elevated temperature was recorded 24 h after the first vaccination in all groups (Fig. 2), the elevated temperature did not reach the reference value for fever in piglets (38.8°C) (15). The temperature decreased back to normal values within 48 h postvaccination. No increase in temperature was observed after the second vaccination or at any other time point during the experiment.

Respiratory and behavioral activity was also monitored in experimental animals to detect any change after the vaccine administration. Based on the clinical report, only one of the piglets in the adjuvanted inactivated vaccine group presented an adverse reaction at 30 s postvaccination, showing salivation, depression, and muscle tremors. The piglet was treated immediately, it was wetted with cold water, and its response was evaluated. After 5 min of the adverse reaction, the pig had no serious clinical manifestations, it remained depressed for 1 h and returned to its normal behavior. This same pig did not show any type of postvaccination adverse reaction after the booster immunization. No other piglets from any of the groups showed clinical manifestations in the daily checkups throughout the study.

Clinical signs were recorded at 30 s; 5, 10, 15, 30, and 60 min; 3, 5, 8, 24, and 48 h and every 24 h after each vaccination until the end of the experiment. Each morning, study animals were monitored for clinical signs compatible with adverse reactions, such as anaphylactic shock, lesions at the vaccination site, or behavior different from the normal one of pigs at their age. All animals were healthy, without signs of abnormal breathing, change in behavior, or changes in rectal temperature. For animal welfare reasons, the animals were observed more than once a day.

At day 35, the pigs were humanely euthanized, and necropsies were performed. It was determined that none of the animals showed lung lesions suggestive of viral infection which could have been caused by the vaccine vector. In the area where the vaccine was applied via the IM route, no active or chronic inflammatory processes were detected, and no fibrosis or abscesses were found. This indicates that the IM or IN application of the vaccine did not cause lesions in the lung or local tissue in the area of the vaccine administration. Based on the results described above, we conclude that there were no severe adverse reactions in the groups vaccinated with the AVX/COVID-12-HEXAPRO, meeting the established safety/adverse reaction parameters (16).

We attempted to isolate infectious NDV in postvaccination nasal swabs, but no viral shedding was detected. Using a quantitative real-time PCR (qRT-PCR), we also sampled the piglets for NDV genomic material as substitute for viral shedding, both at baseline and 24 h after the first vaccination and after the second vaccination. Baseline and postprime samples were found to be negative. However, in the postboost samples of groups 1 to 9 a small amount of genetic material was detected. However, quantification of the genetic material failed, likely due to too-small amounts of RNA.

### AVX/COVID-12-HEXAPRO induces strong antibody responses.

**FIG 3:**
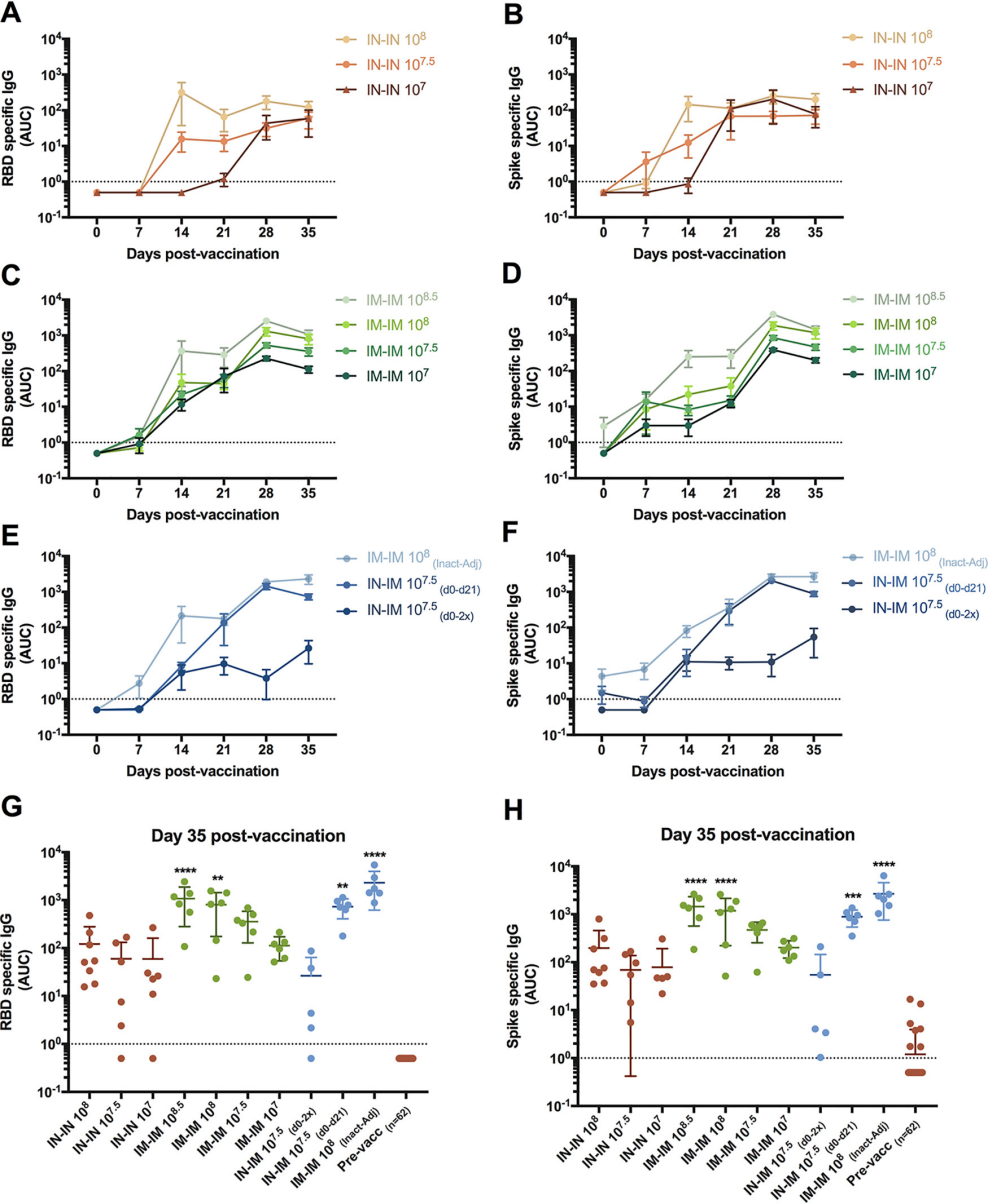
Antibody responses against SARS-CoV-2 RBD and full-length spike ectodomain in sera from vaccinated pigs. Antibody responses against the receptor binding domain (RBD; panels A, C, E, and G) and the full-length spike protein ectodomain (panels B, D, F, and H) were assessed by ELISA. Responses were measured in the IN-IN groups (A and B), in the IM-IM groups (C and D), and in other vaccination regimens (E and F) at 0, 7, 14, 21, 28, and 35 days after the first vaccination. A comparison of individual points for every pig is shown in panels G and H. In panels G and H, d0-2x refers to the administration of the IN and IM doses simultaneously at day 0, and d0-d21 refers to the IN and IM doses administered at days 0 and 21, respectively. Mean of antibody levels per group expressed as area under the curve (AUC) plus standard error of the mean (SEM) is shown. Ordinary one-way analysis of variance (ANOVA) with Dunnett’s multiple-comparison test was applied to panels G and H. All adjusted *P* values of <0.05 were considered statistically significant with a confidence interval of 95%. **, *P* < 0.0011; ***, *P* < 0.0002; ****, *P* < 0.0001.

In order to assess immune responses to the spike protein, we first performed enzyme-linked immunosorbent assays (ELISAs) against both the receptor binding domain (RBD; a prominent target of neutralizing antibodies) and the full-length spike protein ectodomain (which includes the RBD but harbors many additional epitopes) (17, 18). All groups elicited antibody response to the RBD. For the IN-vaccinated animals that received the two higher doses (10^8^ and 10^7.5^ EID_50_), titers increased substantially after the first administration but did not increase much after the boost (Fig. 3A). For the group IN vaccinated with 10^7^ EID_50_, antibodies were detected only after the boost, while no response was observed after the first shot. Animals who received two sequential doses IM showed an antibody response in a dose-dependent manner with clear evidence of an increase after the booster dose (Fig. 3C). Animals which received the IN followed by IM prime-boost regimen as well as animals receiving the adjuvanted inactivated vaccine responded well to both first dose and boost (Fig. 3E). The group receiving the IN and IM vaccine at the same time but no booster dose showed a relatively low response. Very similar kinetics were observed for titers to the full-length spike protein ectodomain (Fig. 3B, D, and F).

When comparing antibody levels for both RBD and spike proteins in all groups on day 35 (14 days postboost), it became clear that the IN-IN groups showed a lower serum IgG response to vaccination than did the IM-IM group (Fig. 3G and H). The dose dependence for the IM-IM groups was clear; for IN-IN there was no clear dose dependence based on geometric mean titers (GMTs), but the response became more homogenous when the dose was increased. Giving the vaccine once via the IM and IN routes at the same time did not result in high titers. However, an IN first administration followed by an IM boost (10^7.5^ EID_50_) resulted in titers comparable to the highest (10^8.5^-EID_50_) IM dose given twice. Inactivated, adjuvanted vaccine given twice also resulted in very high titers.

### The induced antibody responses block angiotensin-converting enzyme (ACE)–RBD interactions.

**FIG 4:**
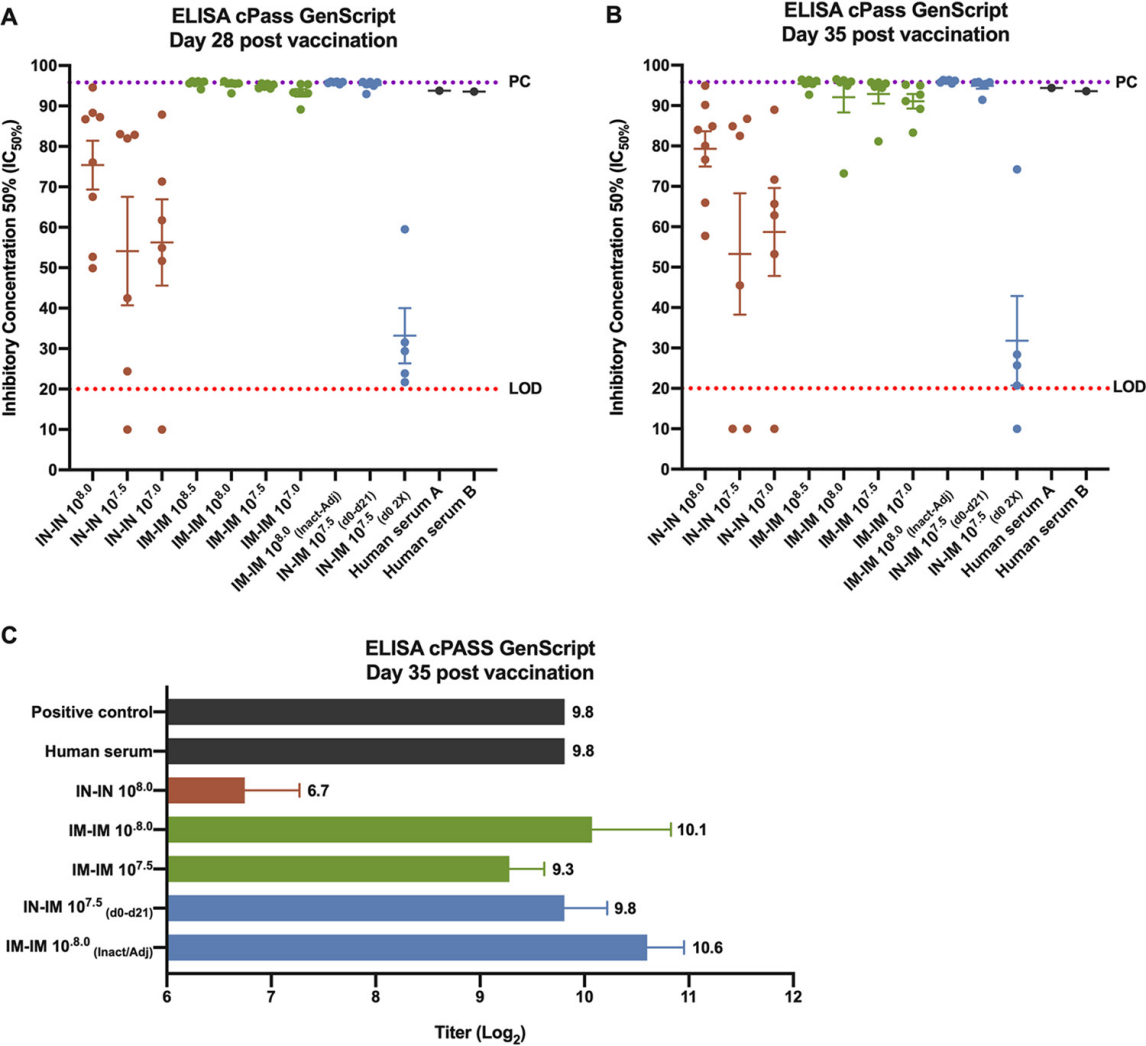
Inhibition of ACE2-RBD interaction by vaccine-induced antibodies. The inhibition of the interaction between the RBD and the receptor angiotensin-converting enzyme 2 (ACE2) was assessed using the ELISA cPass GenScript kit. The 50% inhibitory dilution (ID_50_) using day 28 (7 days postboost) and day 35 (14 days postboost) samples at a single serum dilution (1:20) is presented (A and B). Samples with titers above the upper level of quantification were serially diluted and retested (C). High-titer COVID-19 convalescent-phase human sera were included as additional positive control. For panels A and B, the limit of detection of the assay (LOD) is indicated with the red dotted line, the positive-control range is indicated with the purple dotted line, and the mean of the ID_50_ per group plus standard error of the mean (SEM) is shown. In panel C, titers (log_2_) are presented.

To study the functionality of the antibody response, we first used an assay to measure inhibition of the interaction between the RBD and the receptor ACE2 (19). Initially, all sera were assessed on day 28 (7 days postboost) and day 35 (14 days postboost) at a single serum dilution (1:20). For the IN-IN groups inhibition was detected on day 28, but only one animal reached inhibition close to 100% (Fig. 4A). In contrast, all animals in the IM-IM groups as well as in the IN-IM group and the inactivated adjuvanted group reached levels close to 100%. The group which received the IN and IM vaccine at the same time but only once showed barely any activity. Titers were similar on day 35 with some more variability (Fig. 4B). Since animals in many groups were at the upper level of quantification, sera from these animals were serially diluted and retested. The highest IN-IN dose group approximately matched the titers observed for the lowest IM-IM dose group (Fig. 4C). A clear dose response was observed for the IM-IM groups, and the highest IM-IM dose group and the inactivated adjuvanted group showed indeed the highest titers. Interestingly, the IN-IM (10^7.5^-EID_50_) group also showed a relatively high titer, exceeding the matched 10^7.5^-EID_50_ IM-IM group. The sera from vaccinated animals blocked the ACE2-RBD interaction similarly to a high -titer serum from a convalescent COVID-19 patient (Fig. 4A to C).

### Vaccine-induced antibody responses neutralize SARS-CoV-2.

**FIG 5:**
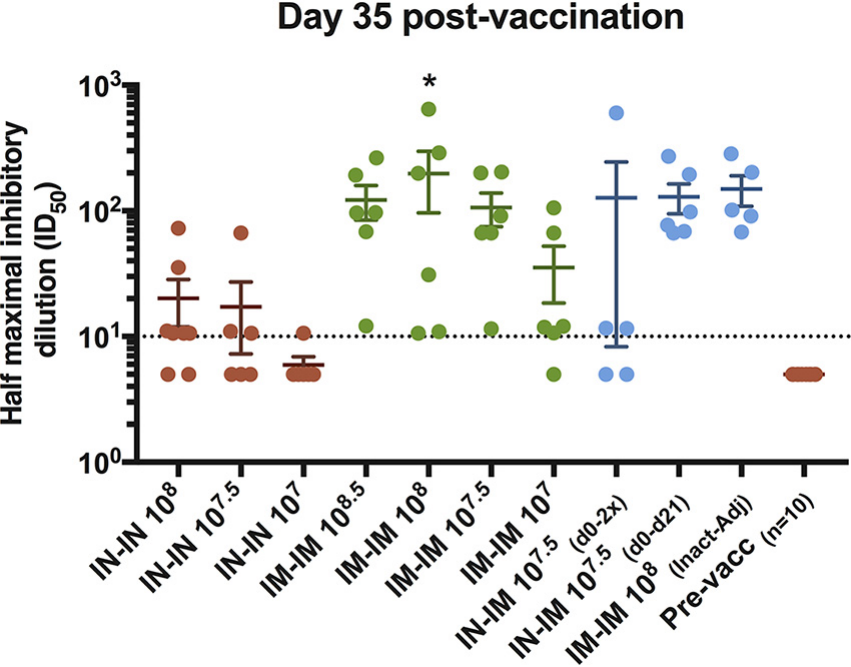
Neutralization of USA‐WA1/2020 SARS-CoV-2 by antibodies in sera from vaccinated pigs. The neutralization activity of the antibodies contained in sera from vaccinated pigs from all the groups was assessed against the authentic USA‐WA1/2020 SARS-CoV-2 using day 35 postvaccination samples. IN-IN vaccinated animals are shown in brown, IM-IM in green, and other vaccination regimens in blue. Neutralization capacity is expressed as the half-maximum inhibitory dilution (ID_50_). Mean of the IC_50_ per group plus standard error of the mean (SEM) is shown. Ordinary one-way ANOVA with Dunnett’s multiple-comparison test was performed. All adjusted *P* values of <0.05 were considered statistically significant with a confidence interval of 95%. *, *P* < 0.0138.

While inhibition of the interaction between RBD and ACE2 can be one mechanism of neutralization, other domains of the spike, especially the N-terminal domain (NTD), are also a target of neutralizing antibodies (20, 21). To determine the neutralizing activity of vaccine-induced sera, we performed neutralization assays with infectious SARS-CoV-2 (22). Animals vaccinated with the two higher IN-IN doses had sporadic neutralizing activity while no neutralizing activity was found in the lowest-dose group (Fig. 5). In contrast, neutralizing activity in the IM-IM groups was more consistent, but some variability was detected in these groups, too. The animals which had received an IM and IN vaccination at the same time but only once had—except for one animal—low titers or no activity. The IN followed by IM group and the inactivated adjuvanted vaccine group had homogenous and relatively high titers. Of note, we also measured hemagglutination inhibition (HI) titers for the NDV vector in all groups over the different time points, which are indicative of the presence of antibodies capable of binding to the hemagglutinin-neuraminidase (HN) protein of the vector virus. We found that titers appeared to trend well with immunity to SARS-CoV-2 spike (see Fig. S1 in the supplemental material). Particularly, the IM-vaccinated groups showed a very good response against the NDV vector after the second dose of the vaccine in a dose-dependent manner, and similar results were obtained for the IN-IM (day 0 [d0] to d21) and IM-IM inactivated adjuvanted groups, while the IN groups exhibited lower HI titers.

10.1128/mBio.01908-21.1 FIG S1 NDV hemagglutination inhibition titers of sera from vaccinated pigs. The hemagglutination inhibition titers for the NDV vector using pig sera from all the experimental groups collected at 0, 7, 14, 21, 28, and 35 days postvaccination are presented. The cutoff of the assay (5.31 log^2^) is indicated with a red dotted line. Download FIG S1, TIF file, 0.8 MB. Copyright © 2021 Lara-Puente et al. 2021 Lara-Puente et al. https://creativecommons.org/licenses/by/4.0/ This content is distributed under the terms of the Creative Commons Attribution 4.0 International license.

### AVX/COVID-12-HEXAPRO induces antibodies in pigs that recognize spike proteins of variants of concern.

**FIG 6:**
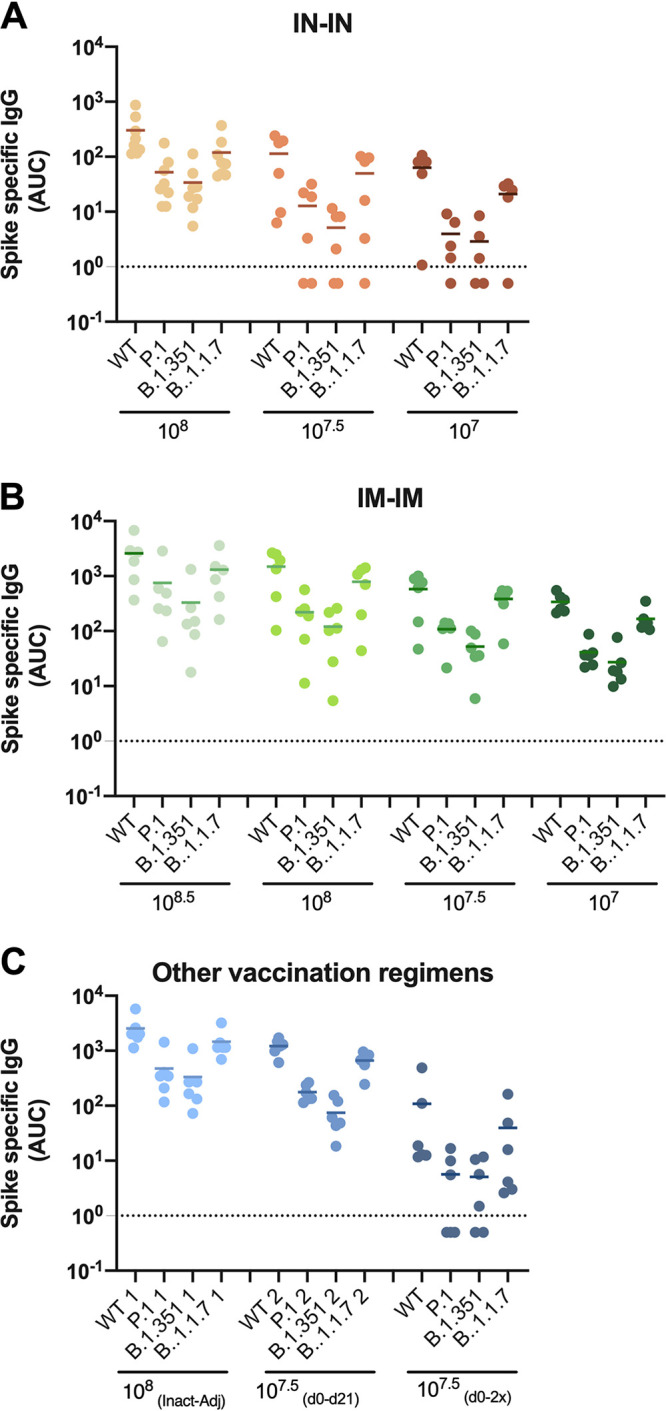
Cross-reactivity of vaccine-induced antibodies to the spike protein of variants of concern. The reactivity of vaccine-induced antibodies was assessed by ELISA against the variants of concern P.1, B.1.1.7, and B.1.351 compared to the wild-type (WT) spike protein. IN-IN vaccinated animals are shown in panel A, IM-IM in panel B, and other vaccination regimens in panel C. Mean of antibody levels per group expressed as area under the curve (AUC) is shown.

Finally, we also assessed the cross-reactivity of vaccine-induced antibodies to the spike protein of variants of concern B.1.1.7 (23, 24), B.1.351 (25, 26), and P.1 (27) in an ELISA and compared it to wild-type SARS-CoV-2 spike. No substantial drop in reactivity was detected against B.1.1.7 (Fig. 6) compared to wild type. A decrease in reactivity was detected for P.1 and B.1.351 in all groups (Fig. 6). However, reactivity was well maintained in the high-dose IN-IN group, in all IM-IM groups, and in the IN-IM group as well as in the inactivated, adjuvanted group (Fig. 6). Coating efficiencies of the different antigens were similar (Fig. S2), allowing for a direct comparison.

10.1128/mBio.01908-21.2 FIG S2 Coating efficiency of the different antigens in the ELISAs against spike proteins from variants of concern. ELISA plates were coated with P.1, B.1.1.7, B.1.351, or WT spike proteins. Plates were incubated with an anti-6×His antibody for detection of the specific protein tag. Antibody levels expressed as area under the curve (AUC) are shown. Download FIG S2, TIF file, 0.1 MB. Copyright © 2021 Lara-Puente et al. 2021 Lara-Puente et al. https://creativecommons.org/licenses/by/4.0/ This content is distributed under the terms of the Creative Commons Attribution 4.0 International license.

## DISCUSSION

Several excellent vaccines against SARS-CoV-2 are currently in use, but production capacity is insufficient to satisfy the global demand. Here, we tested a novel vaccine candidate, AVX/COVID-12-HEXAPRO, also known as Patria, in a pig model. This candidate is an NDV-based live vectored vaccine candidate, and this vaccine platform has several advantages. The vaccine can be produced at low cost in embryonated eggs in established facilities that are used to produce influenza virus vaccines. NDV grows very well in that substrate, and a single embryonated egg yields several vaccine doses. NDV is an avian virus that is harmless for humans. It is currently in clinical trials as an oncolytic agent where it is used at very high doses in cancer patients and has—even in these immunocompromised individuals—been shown to be safe (12, 13, 28). One reason why NDV is not pathogenic in mammals is because it triggers interferon responses, which not only restrict its growth in the mammalian host but also have an adjuvating effect on adaptive immunity, leading to stronger adaptive immune responses (9, 10). Furthermore, humans have no preexisting immunity to this vector. Finally, intranasal administration triggers mucosal immune responses locally in the respiratory tract, which can be highly protective because they can stop the virus at its entry point. In addition to these platform-dependent advantages, the AVX/COVID-12-HEXAPRO also features an optimized spike antigen, based on the HexaPro construct (14) that is stabilized by the introduction of 6 prolines (compared to introduction of two prolines in the mRNA and some vectored vaccines [29–33]). The HexaPro construct has been further optimized by adding the transmembrane and cytoplasmic domains of the NDV fusion protein to enhance its expression on the surface of the NDV particles.

Here, we have chosen to test this vaccine candidate in a pig model. Earlier versions of the vaccine have also been tested in rodents as inactivated and live vaccines (5, 6). However, pigs are a large-animal model that physiologically resembles humans well and are often used for safety studies (34). In addition, while pigs do not support the replication of SARS-CoV-2 well (35), they are the natural host for alphacoronaviruses such as transmissible gastroenteritis virus (TGEV [36]), porcine epidemic diarrhea virus (PEDV [37, 38]), porcine respiratory coronavirus (PRCV [36]), and swine acute diarrhea syndrome coronavirus (SADS-CoV [39]); betacoronaviruses (porcine hemagglutinating encephalomyelitis virus (PHEV [40]); and the deltacoronavirus porcine deltacoronavirus (PDCoV [41, 42]). This study uses specific-pathogen-free (SPF) pigs to avoid any interference from cross-reactive immune responses against porcine paramyxoviruses or coronaviruses. Importantly, the safety and immunogenicity of recombinant virus-based vaccines administered via the IM or IN routes have been assessed in the pig model. For example, virus-vectored Nipah virus vaccines administered via the IM route are able to induce long-lasting neutralizing antibody titers in this model (43). Recombinant NDV vaccines based on the LaSota strain—the same strain used in our study—expressing proteins from the highly pathogenic porcine reproductive and respiratory syndrome virus (PRRSV) not only had a good safety profile but resulted in induction of robust neutralizing antibody responses after IM administration (44). Likewise, recombinant NDVs expressing immunogens of the classical swine fever virus given via the IN route were safe and immunogenic in pigs (45). Overall, these data and our results support the safety profile and immunogenic potential of recombinant virus-based vaccines, particularly recombinant NDV.

We found that the vaccine candidate was safe in this model, independently of the route of administration or dose delivered. However, immunogenicity varied depending on the dose and vaccination route. IN vaccination induced relatively low antibody titers (binding as well as functional) in serum. Intramuscular vaccination led to strong antibody responses in serum in a dose-dependent manner. A combination of the two approaches—the vaccine given intramuscularly and intranasally at the same time once—failed to induce robust immunity. However, when a prime-boost regimen was used with an IN administration and an IM boost, the titers measured in serum were similar to those for intramuscular vaccination. Importantly, this regimen likely induces mucosal immunity (7, 16, 45) and therefore likely results in both upper respiratory tract protection (mucosal immunity mostly mediated by secretory IgA) and lower respiratory tract protection (systemic immunity mostly mediated by IgG). Finally, we also evaluated an inactivated, adjuvanted vaccine formulation which induced strong antibody responses in serum as well. Importantly, the antibody responses induced by AVX/COVID-12-HEXAPRO were functional and inhibited the interaction between ACE2 and the viral RBD and neutralized authentic SARS-CoV-2. The emergence of variant viruses that can partially escape antibody responses is of concern. We tested the reactivity of antibodies induced by AVX/COVID-12-HEXAPRO in pigs for cross-reactivity to the B.1.1.7, B.1.351, and P.1 variants. We found little difference in binding to B.1.1.7 spike protein, and while binding was reduced to B.1.351 and P.1 proteins, animals in most groups still had robust reactivity to those spike proteins. We hypothesize that the optimized antigen design of AVX/COVID-12-HEXAPRO contributes to this cross-reactivity, favoring the induction of neutralizing antibodies to multiple S protein epitopes. It remains to be determined if serum from humans vaccinated with AVX/COVID-12-HEXAPRO shows similar cross-reactivity. Of note, if a variant arises that warrants a change in vaccine strains or the formulation of a multivalent vaccine, this could be easily achieved with the NDV platform described here.

In this study, we tested a live version of our NDV-based SARS-CoV-2 vaccine approach. The SARS-CoV-2 spike is not only present on the surface of the NDV particles but also expressed on the surface of cells infected by the NDV-vectored vaccine, which should lead to enhanced T-cell responses. Using a live NDV vector, as discussed above, also allows intranasal delivery and potential induction of mucosal immune responses. In parallel, we are also developing, in collaboration with PATH, Instituto Butantan in Brazil (NCT04993209, ButanVac), the Government Pharmaceutical Organization (GPO) in Thailand (NCT04764422), and the Institute of Vaccines and Medical Biologicals (IVAC) in Vietnam (NCT04830800, COVIVAC), a different, inactivated version of the NDV-based vaccine termed NDV-HXP-S. Current clinical trials in Thailand, Vietnam, and Brazil include adjuvanted and/or nonadjuvanted formulations of these inactivated vaccines. As seen in this study, an inactivated adjuvanted formulation of the vaccine was highly immunogenic and safe and represents an alternative clinical development path.

This study also has limitations. First, we were not able to evaluate the immune response locally in the nasal mucosa or the respiratory tract of vaccinated pigs, since appropriate sampling methods and standardized tests to assess immunity in the mucosa were not yet established and qualified locally. Also, no challenge was performed, since the pig model is not susceptible to SARS-CoV-2, but this vaccine has been tested in other animal models susceptible to challenge studies with excellent results (5, 6).

Based on the results obtained from this animal study, a clinical trial has been initiated in Mexico City, Mexico, with AVX/COVID-12-HEXAPRO (NCT04871737). This trial features IN-IN, IM-IM, and IN-IM groups with different doses of the live AVX/COVID-12-HEXAPRO vaccine candidate and allows assessment of safety and immunogenicity of this vaccine in humans. If the vaccine proves to be safe and efficacious in clinical trials, it could significantly contribute to satisfying the global need for SARS-CoV-2 vaccines. The cost-effective and simple production process—which is available in many countries including low- and middle-income countries (46)—makes this vaccine candidate an affordable and sustainable solution for the prevention of COVID-19.

## MATERIALS AND METHODS

### Ethics statement.

Animal studies were performed in accordance with Laboratorio Avi-Mex, S. A. de C. V., standard operating procedures (SOP) and quality policies (Comité Institucional para el Cuidado y Uso de los Animales de Laboratorio [CICUAL/UNAM] OFICIO/FQ/CICUAL/428/21). Inspections of the protocol, animal facilities, or both are performed by a representative designated by the company during the study in accordance with the appropriate SOP. The study was conducted in accordance with the Animal Welfare Act (9 CFR Subchapter A 1–4) and other standards appropriate for the study, such as the international harmonization of technical requirements for the registration of veterinary products (Veterinary International Conference on Harmonization [VICH]) and requirements of good clinical practice (GCP), VICH GCP Principles (VICH GL55 [Biologicals: Target Animal Batch Safety Testing Live Vaccines, May 2017]). The animals were randomly placed in biosafety level 2 (BSL-2) biocontainment isolation cubicles according to the experimental design. After 35 days since the first vaccination (days postvaccination [DPV]), animals were euthanized by electrically induced unconsciousness and exsanguination was performed by cutting the brachial plexus. During the necropsy, samples of tonsil, lymph nodes, lung, liver, kidney, and spleen were taken from each animal from all groups.

### Experimental vaccine.

The experimental active AVX/COVID-12-HEXAPRO vaccine was provided frozen, in vials of 30 ml per 12 (IM) doses or 12 (IN) doses, depending on the route of application.

### Vaccine manufacturing.

The AVX/COVID-12-HEXAPRO vaccine was grown in 10-day-old SPF chicken embryos by inoculation via the allantoic cavity, with 10^3.3^ EID_50_/0.1 ml of the production seed. The embryos were incubated for a period of 72 h at an average temperature of 37°C and a range of 60% to 70% relative humidity. After this period, the embryos were sacrificed by refrigeration for at least 12 h. Harvesting of the allantoic fluid (AF) was carried out under aseptic conditions, by vacuum. The AF was clarified by means of filters with a pore size of 0.8 to 8.0 μm, concentrated by a factor of 10× in 300-kDa cassettes, and subjected to diafiltration in 20 volumes of phosphate-buffered saline (PBS). AF was stored frozen at −70°C.

The AF resulted in a titer of 10^8.67^ EID_50_/ml, and from it, the preparation of the frozen active vaccines was made. The corresponding volumes of purified AF were aseptically mixed with the stabilizer TPG (trehalose, monobasic potassium phosphate, dibasic sodium phosphate, monosodium glutamate) and were mixed homogeneously for 5 min using a Teflon bar and a magnetic stirrer. Finally, they were packed in high-density polyethylene bottles. The vaccines were stored at −80°C.

### Inactivated vaccine.

For the inactivated vaccine, the diafiltrated AF was inactivated by adding formaldehyde to a final concentration of 0.037% (vol/vol). The inactivation was carried out by adding the 3.7% formaldehyde solution in PBS dropwise to the AF under constant stirring. After adding the formaldehyde solution, it was kept for 16 h at 37°C under continuous stirring. Finally, the fluid was refrigerated at 2°C to 4°C.

The adjuvant water oil water (WOW) vaccine was prepared by adding the inactivated fluid (13 ml) to the previously prepared WOW emulsion (47 ml) and mixing in a vortex for 3 min. The inactivated vaccine was formulated to provide an equivalent of 10^8.0^ EID_50_/ml, and the vaccine was kept refrigerated. The adjuvant used was Inmunopromt (Laboratorio Avi-Mex, S. A. de C. V., proprietary).

The WOW emulsion was previously prepared by formulating separately the oil phase, the internal aqueous phase, and the external aqueous phase. First, a water-in-oil (WO) emulsion was prepared by adding the external aqueous phase to the oil phase with continuous stirring. For the preparation of the WOW emulsion, the external aqueous phase was placed in a blender and, while operating the same, the WO emulsion was added. Stirring was continued for 3 min. At the end of the stirring, the mixture was packaged and stored in refrigeration.

### Animal testing.

A total of 62 specific-pathogen-free (SPF) pigs (Avifarma, S. A. de C. V., Mexico) of similar body weight per group (±1.5 kg), between 3 and 10 weeks of age, were used in the study for the evaluation of AVX/COVID-12-HEXAPRO in the different experimental groups. The animals were randomly placed in the cubicles upon arrival. Treatment group assignments were known only to those who administered the doses. SPF pigs free of all major porcine pathogens including PRRSV, swine influenza virus (SIV), TGEV, PEDV, PDCoV, and Mycoplasma hyopneumoniae were selected for the study. All animals used for this study were the property of the study unit, and by signature of the investigator in the protocol, permission was granted for their use in the study. Piglets were not vaccinated after birth and were not treated with any drugs known to interfere with vaccination. Animals were acclimatized for 4 days before the start of the study. Day 0 of the study was the same for all animals and was the day of administration of the experimental AVX/COVID-12-HEXAPRO vaccine in its different formulations and doses. The animals were monitored once a day for clinical signs, from the beginning to the end of the study. Each active formula was evaluated separately and simultaneously.

Animals in the IN-IN groups were administered a dose of 2.0 ml by the intranasal route of the active AVX/COVID-12-HEXAPRO vaccine, with target titers of 10^8.0^ EID_50_ (measured titer 10^8.39^ EID_50_), 10^7.5^ EID_50_ (measured titer 10^8.04^ EID_50_), and 10^7.0^ EID_50_ (measured titer 10^7.58^ EID_50_). This was repeated on day 21 after the first vaccination. Animals in the IM-IM groups were vaccinated on day 0 with a dose of 1.0 ml with a target titer of 10^8.5^ EID_50_ (measured titer 10^8.24^ EID_50_), 10^8.0^ EID_50_ (measured titer 10^7.92^ EID_50_), 10^7.5^ EID_50_ (measured titer 10^7.44^ EID_50_), and 10^7.0^ EID_50_ (target and measured titer), respectively, and were revaccinated on day 21 postprime. One additional group simultaneously received a 2.0-ml intranasal dose and a 1.0-ml intramuscular dose, with a target titer of 10^7.5^ EID_50_ (measured titer 10^8.04^ EID_50_) and target titer of 10^7.5^ EID_50_ (measured titer 10^7.44^ EID_50_), respectively, on day 0 but without a booster vaccination. One group received a 2.0-ml intranasal dose with a target titer of 10^7.5^ EID_50_ (measured titer 10^8.04^ EID_50_) on day 0 and a second (intramuscular) dose of 1.0 ml with a target titer of 10^7.5^ EID_50_ (measured titer 10^7.44^ EID_50_) on day 21. Finally, one group was vaccinated IM using the inactivated AVX/COVID-12-HEXAPRO vaccine with a preinactivation titer of 10^8.0^ EID_50_ in a dose of 1.0 ml and was revaccinated on day 21 after the first vaccination.

The live vaccine was thawed before use and was kept at refrigeration temperature (between 4°C and 8°C) until its application for no more than 2 h once thawed. The inactivated vaccine was adjusted to a temperature of 10°C to 14°C for use and was applied within 2 h after the temperature was raised.

The intranasal vaccination process was carried out with an automatic syringe (Prima bottle mount vaccinator) with a capacity of 2.0 ml per dose for the administration of the 2.0-ml intranasal vaccination, with a patented applicator for intranasal administration in pigs (Prima mist sprayer). Each of the piglets was taken by an operator, who held the animal by the back, placing it in a vertical position in front of the vaccinating operator. The vaccine was administered through the intranasal applicator, and the operator tried to match the administration with an inhalation by the piglet. After one more inhalation, the piglet was released, and vaccination proceeded with another animal. A separate applicator was used for each group. For the intramuscular vaccination process, a syringe with a sterile 22 × 1-gauge needle was used. The automatic syringe calibration was in this case 1.0 ml/dose. Following the previous animal handling procedure, the operator held the piglet horizontally to gain access to the dorsocranial part of the piglet’s neck. Antisepsis using a cotton pad with 70% ethyl alcohol was performed in the vaccination area, and the vaccine was applied (deeply) intramuscularly in the anterior part of the loin muscle. One needle and syringe were used for every group of vaccinated animals, and thereafter, the needle and the syringe were changed for a new sterile one.

### Evaluation of clinical signs and safety evaluation.

Animals were observed for clinical signs throughout the period of acclimatization, study, and vaccine administration and continuing through necropsy. Each study animal was monitored for clinical signs or abnormal breathing, abnormal attitude, and rectal temperature each morning. For animal welfare reasons, the animals were observed more than once a day.

General health observations were conducted by a veterinarian, or someone designated, duly qualified, and were recorded daily starting with the day of arrival and until the end of the study. Any abnormal health observations or activity were documented. All observations of health abnormalities after inoculation were documented (clinical evaluation).

The following was used as a guide for a classification of the assignment of a clinical score for clinical signs or abnormal respiratory attitude. The clinical score was defined as similar or normal to healthy animals at the site. Care was taken in assessing differences between normal animals and distinguishing between normal physiological responses and pathological responses.

### Respiratory evaluation.

Respiratory status was graded as follows: 0, normal (chest breathing with some abdominal movement); 1, mild respiratory distress (some abdominal breathing); 2, moderate respiratory distress (exaggerated and laborious abdominal breathing); 3, severe respiratory distress (very laborious breathing, abdominal breathing; open mouth, cyanosis of the nose and ears).

### Activity/depression assessment.

Activity status was graded as follows: 0, normal (pigs react/growl when opening the door; pigs are active, playful, and curious; they look at the door, approach it, and smell it; they show interest in food and water; if they get excited, they can urinate and defecate); 1, mild (they get up when stimulated but slowly and with not much interest or curiosity, and they go back to bed fast; there is some interest in food); 2, moderate (pronounced inactivity and reluctance to get up/move; there is prostration, incoordination); 3, severe (there is no response; they do not get up).

There were no clinical manifestations evident in the daily checkups in any of the piglets of all groups, throughout the development of the test (except for the one pig which had a brief reaction after administration of the inactivated/adjuvanted vaccine).

This indicates that the vaccines used, with different formulations and route of administration, were safe and met the safety test (47).

During the vaccination process, all animals were clinically evaluated in search of signs compatible with adverse reactions, such as anaphylactic shock, lesions at the vaccination site, or behavior different from that of normal same-age pigs. Data were recorded at 30 s; at 5, 10, 15, 30, and 60 min; at 3, 5, 8, 24, and 48 h; and every 24 h after each vaccination and until the end of the test.

### Pathology tests.

Fourteen days after the second vaccination (day 35 after the first dose), all animals were humanely euthanized and lung, lymph node, liver, kidney and spleen samples were collected, to determine the presence of the vaccine virus by qRT-PCR and to perform histopathology analysis. An experienced pathologist took photographs and carried out the evaluation of possible lung lesions using the planimetry technique for scoring (48) and evaluated the changes of the lung at the macroscopic and microscopic level following the intranasal administration of the vaccine. A histopathological evaluation was also done in the areas of the muscular applications of the vaccine.

### Viral load.

To determine the viral load, samples were taken (nasal swabs on day 0 prevaccination, 1 day after first vaccination, and 1 day after second vaccination). Samples were analyzed via qRT-PCR targeting the vector and the spike insert to evaluate the presence of and viral load for the vaccine vector. For that purpose, we used a commercial kit, VetMAX NDV reagents (Applied Biosystems, catalog number 4406874); the positive and negative controls used were from TaqMan Newcastle disease virus (NDV) and Xeno RNA (Applied Biosystems, catalog number 4406875), and all the reactions were run in an Applied Biosystems 7500 Fast thermocycler, following the instructions of the manufacturer. The viral load was also evaluated after necropsy in lung tissue samples.

### Cells and recombinant proteins.

Vero.E6 cells (ATCC catalog no. CRL-1586) cells were maintained in culture using Dulbecco’s modified Eagle’s medium (cDMEM; Gibco) supplemented with 10% fetal bovine serum (FBS; Corning) and an antibiotic solution containing 10,000 units/ml of penicillin and 10,000 μg/ml of streptomycin (Pen-Strep; Gibco). Recombinant proteins corresponding to the receptor binding domain (RBD) of SARS-CoV-2 and full-length ectodomain spike were produced in-house using the HEK 293F mammalian cell expression system and purified as previously described in much detail (18).

### Enzyme-linked immunosorbent assays (ELISAs).

Pigs were bled 0, 7, 14, 21, 28, and 35 days after the first vaccine dose administration. Antibodies in pig sera against the receptor binding domain (RBD) or spike (S) were measured as previously described (17, 18). Briefly, polystyrene 96-well plates (Immulon 4HBX; Thermo Fisher Scientific) were coated with 50 μl/well of PBS (pH 7.4) (Gibco) containing recombinant RBD or spike proteins (2 μg/ml) and incubated at 4°C overnight. On the next day, plates were washed with PBS-0.1% Tween 20 (PBS-T) using an automated plate washer (AquaMax 2000; Molecular Devices). Plates were blocked with 220 μl/well of PBS-T, 3% nonfat dry milk (AmericanBio) for 1 h. For serum and secondary antibody dilutions, a solution of PBS-T, 1% nonfat dry milk (AmericanBio) was used. Sera were serially diluted (3-fold) starting at a 1:100 dilution. Dilutions were added to the plates (100 μl/well) for 2 h at room temperature (RT). Plates were washed, and the secondary antibody anti-pig IgG (whole molecule)-peroxidase antibody produced in rabbit (50 μl/well, Sigma no. A5670) was added at a 1:6,000 dilution for 1 h at RT. Plates were washed, and the substrate *o*-phenylenediamine dihydrochloride (SigmaFast OPD; Sigma-Aldrich) was added (100 μl/well) and incubated for 10 min. The reaction was stopped by addition of 50 μl/well of a 3 M HCl solution (Thermo Fisher Scientific). Optical density (OD) was measured (490 nm) using a microplate reader (Synergy H1; BioTek). Analysis was performed using Prism 7 software (GraphPad), and values were reported as area under the curve (AUC).

### ACE2-RBD interaction inhibition assay.

For the evaluation of antibodies that inhibit the interaction between ACE2 and the RBD, a commercial kit, cPass SARS-CoV-2 neutralization antibody test (GenScript), was used according to the manufacturer’s instructions. The GenScript SARS-CoV-2 surrogate virus neutralization test (sVNT) kit can detect circulating neutralizing antibodies against SARS-CoV-2 that block the interaction of the receptor binding domain of the spike glycoprotein (RBD) with the ACE2 cell surface receptor. The assay detects any antibodies in serum and plasma that neutralize the RBD-ACE2 interaction. The test is both species and isotype independent.

The SARS-CoV-2 sVNT kit is a blocking ELISA detection tool, which mimics the virus neutralization process. The kit contains two key components: the horseradish peroxidase (HRP)-conjugated recombinant SARS-CoV-2 RBD fragment (HRP-RBD) and the human ACE2 receptor protein (hACE2). The protein-protein interaction between HRP-RBD and hACE2 can be blocked by neutralizing antibodies against SARS-CoV-2 RBD.

We used the negative and positive controls included in the kit for validation and interpretation of the test.

Initial samples were tested at a set 1:20 dilution. For select groups, dilution series were performed and assayed as well.

### Microneutralization assay.

Pig sera collected 35 days after the first vaccine dose administration were used to assess the neutralization of SARS-CoV-2 USA‐WA1/2020, as previously described (22). All procedures were performed in the biosafety level 3 (BSL-3) facility at the Icahn School of Medicine at Mount Sinai following standard safety guidelines. All steps during the microneutralization assay, staining, and analysis of data were performed as previously described in detail (22).

### Hemagglutination inhibition (HI) test.

The serum was absorbed with a solution of kaolin and erythrocytes to 5% and incubated overnight at refrigeration temperature (2°C to 8°C). The next day, the sera were centrifuged and diluted in a PBS solution; the dilution started at 1:10 and went to 1:20,480. A Newcastle disease virus solution, to 8 hemagglutinating units (HAU), was added to the diluted sera and incubated 30 min at room temperature (18°C to 24°C). To finish, we added an erythrocyte solution to 0.5% and incubated the mixture for 30 min at RT. The sample was considered positive when the titer obtained was equal to or greater than 1:40 (5.3 log_2_).

### Sample size and statistics.

The sample size was calculated using the G-power program version 3.1.9.4, guaranteeing that the power of the study was greater than 80%. In the case of deaths of animals during the experimental procedures, the power of the study will not be affected because the size of the sample is greater than a power of 80%. All graphics were made and statistical analyses were performed using GraphPad Prism 9.1.0.221 for Windows (GraphPad Software, San Diego, CA, USA).
