# Supplementary figures and images for: Safety and Immunogenicity of a Newcastle Disease Virus Vector-Based SARS-CoV-2 Vaccine Candidate, AVX/COVID-12-HEXAPRO (Patria), in Pigs
Source: mBio. 2021 Sep 21;12(5):e01908-21. doi: 10.1128/mBio.01908-21 (PMC8546847; doi:10.1128/mBio.01908-21)

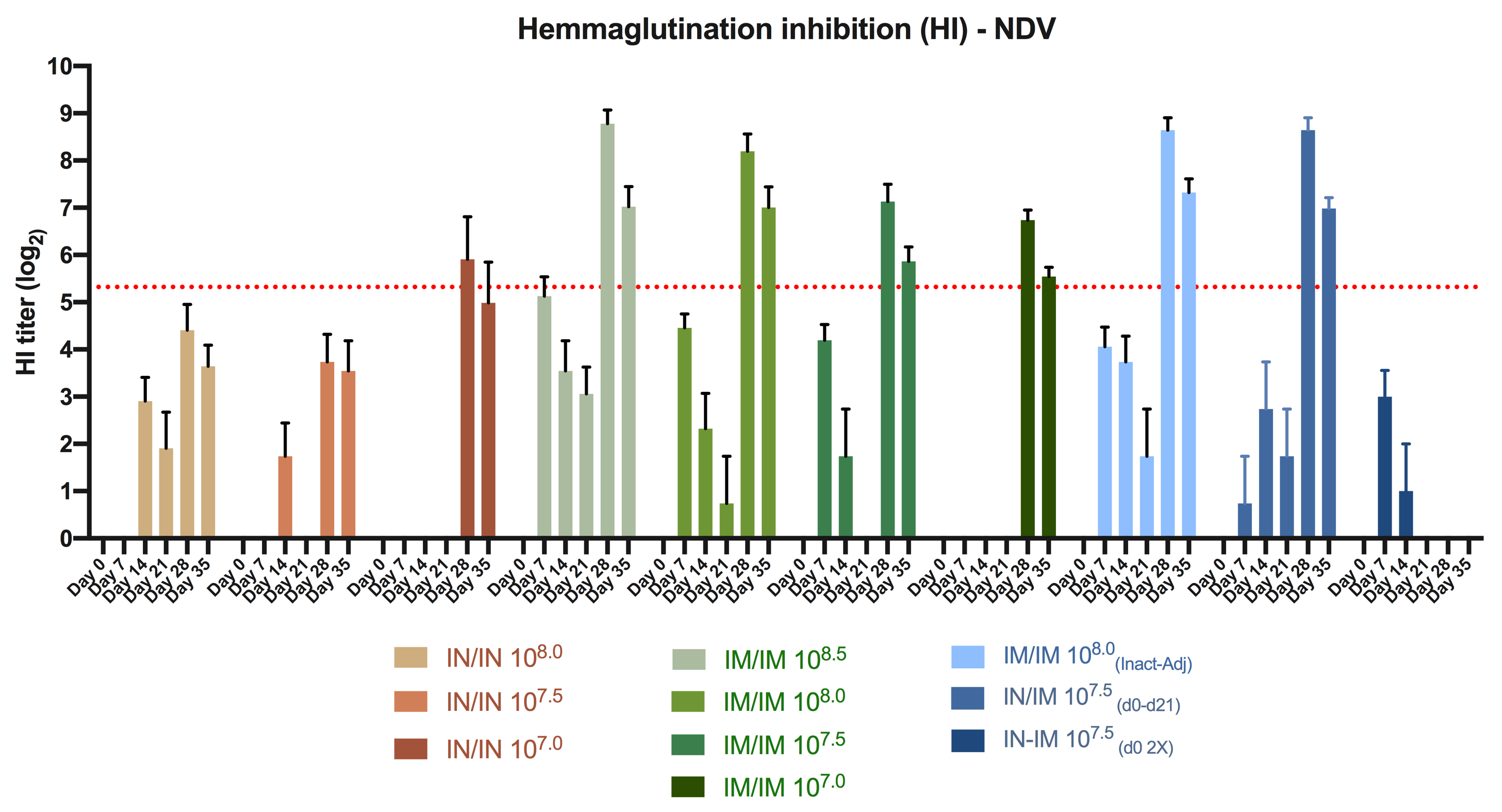

Supplement: FIG S1 [file mbio.01908-21-sf001.tif]

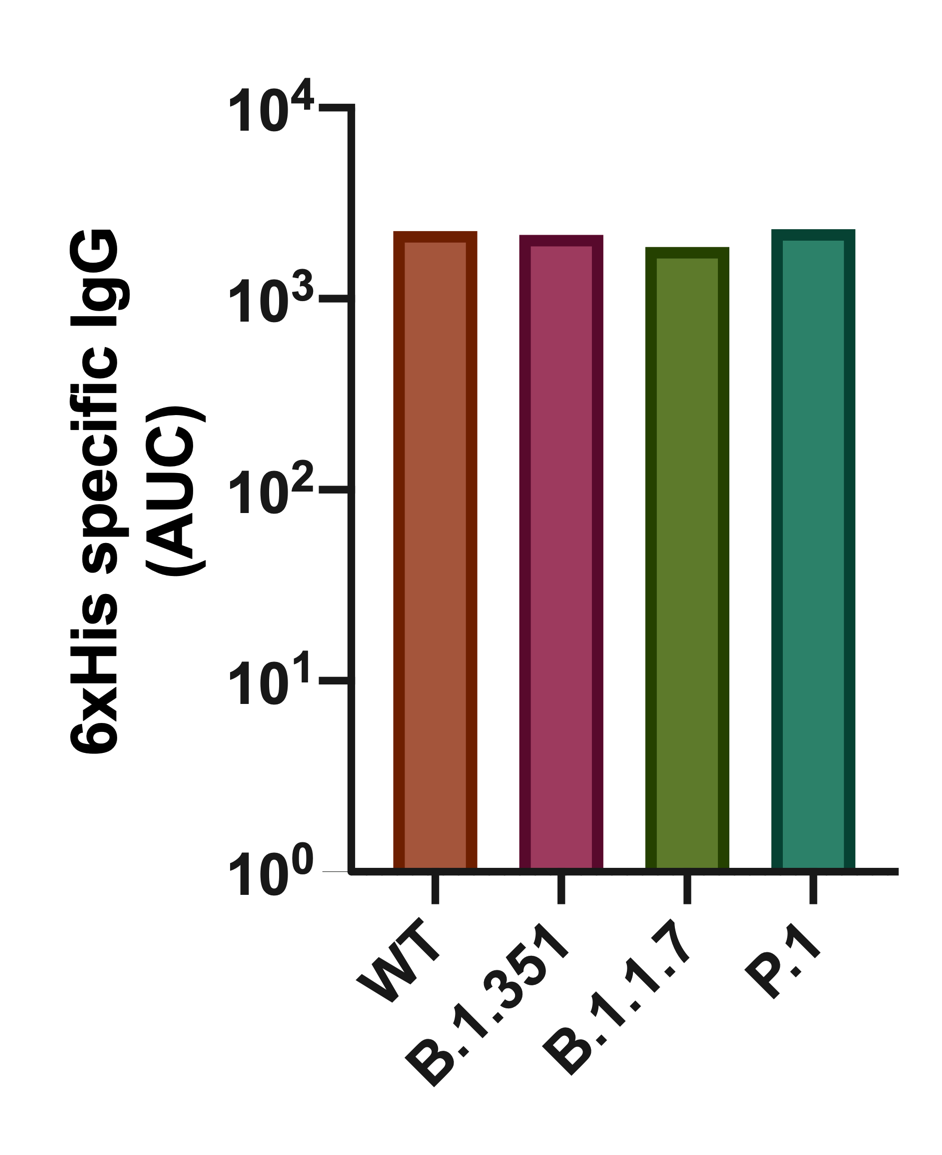

Supplement: FIG S2 [file mbio.01908-21-sf002.tif]
